# Controllable Fabrication of Fe_3_O_4_/ZnO Core–Shell Nanocomposites and Their Electromagnetic Wave Absorption Performance in the 2–18 GHz Frequency Range

**DOI:** 10.3390/ma11050780

**Published:** 2018-05-11

**Authors:** Xiaodong Sun, Guangyan Ma, Xuliang Lv, Mingxu Sui, Huabing Li, Fan Wu, Jijun Wang

**Affiliations:** 1 Key Laboratory of Science and Technology on Electromagnetic Environmental Effects and Electro-Optical Engineering, The Army Engineering University of PLA, Nanjing 210007, China; xiaodongsun1001@hotmail.com (X.S.); xllu1957@126.com (X.L.); plasmx@126.com (M.S.); 2 College of Field Engineering, The Army Engineering University of PLA, Nanjing 210007, China; cgzovezy@163.com; 3 School of Mechanical Engineering, Nanjing University of Science & Technology, Nanjing 210094, China; 4 Research Institute for National Defense Engineering of Academy of Military Science PLA China, Beijing 100036, China

**Keywords:** core–shell structure, electromagnetic absorption, interfacial polarization, Fe_3_O_4_, ZnO

## Abstract

In this study, Fe_3_O_4_/ZnO core–shell nanocomposites were synthesized through a chemical method of coating the magnetic core (Fe_3_O_4_) with ZnO by co-precipitation of Fe_3_O_4_ with zinc acetate in a basic medium of ammonium hydroxide. The phase structure, morphology and electromagnetic parameters of the Fe_3_O_4_/ZnO core–shell nanocomposites were investigated. The results indicated that the concentration of the solvent was responsible for controlling the morphology of the composites, which further influenced their impedance matching and microwave absorption properties. Moreover, Fe_3_O_4_/ZnO nanocomposites exhibited an enhanced absorption capacity in comparison with the naked Fe_3_O_4_ nanospheres. Specifically, the minimum reflection loss value reached −50.79 dB at 4.38 GHz when the thickness was 4.5 mm. It is expected that the Fe_3_O_4_/ZnO core–shell structured nanocomposites could be a promising candidate as high-performance microwave absorbers.

## 1. Introduction

In recent decades, advanced electromagnetic (EM) applications have taken on a fundamental role in areas such as satellite communication, radar systems, and wireless networks [1,2,3,4,5]. However, the problem of powerful electromagnetic interference (EMI) is becoming serious. EMI pollution certainly hinders the extensive utilization of electromagnetic wave (EMW) devices and has many negative effects on the environment and human health. Many efforts have been devoted to investigating efficient solutions for eliminating EMI pollution. Microwave absorption materials (MAMs) are a kind of functional material that can effectively absorb the energy of EMW on their surface and then transform that EMW energy into thermal energy [6,7,8,9]. The development of efficient MAMs is being pursued with high demand, and a considerable number of concepts have been actively investigated in order to develop MAMs with properties including light weight, low price, low thickness, wide absorption bandwidth capability, strong absorption intensity, and anti-oxidation [10,11,12].

Traditional MAMs, including ferrite [13], inorganic metal salts [14], carbonyl iron [15], graphene [16], and conducting polymers [17], have been widely employed in various applications. However, these materials are hardly able to satisfy all of the requirements of qualified MAMs. Typically, permittivity (dielectric property) and permeability (magnetic property) are the key factors influence the absorption property of MAMs. Much research has focused on the synthesis and complementation of different components in order to avoid poor impedance matching [18,19]. According to the EMW absorption mechanism, the microwaves can be absorbed on a large scale and dissipated into thermal energy through magnetic losses and dielectric losses if the characteristic impedance of the absorber is well matched [20]. The composition of magnetic and dielectric materials is significant in improving the impedance matching between permeability and permittivity. Additionally, the employment of different micro-structures in the absorbers could influence their properties. Among the many existing micro-structures, core–shell structures, designed with magnetic components and dielectric components, have attracted a great deal of attention due to their superior microwave absorption properties, which benefit from induced interfacial polarization, as well as improved impedance matching [21]. 

One-dimensional (1D) zinc oxide (ZnO)-related nanomaterials have attracted enormous attention in recent decades as dielectric absorbents because of their light weight and dielectric semiconductive properties [22]. To date, many types of ZnO-based materials have been reported that confirm that the absorption property can be modified by compositing ZnO with magnetic materials [22,23,24]. Additionally, employing ordinary magnetic-dielectric materials as surrogates for rare metals is cost-effective and utilitarian. Considering the composite synthetic technique of ZnO, many progressive methods have been reported in previous works, such as Zn/ZnO [25], Cu/ZnO [25], and reduced graphene oxide/ZnO [26].

Previous studies have confirmed that good EM impedance matching and the efficient complementarity between relative permittivity and permeability can be realized by the synergistic effect of the magnetic and the dielectric compositions. In the present work, taking this principle into consideration, we chose ferroferric oxide as the magnetic counterpart and synthesized Fe_3_O_4_/ZnO core–shell structured nanocomposites with Fe_3_O_4_ cores and ZnO shells. The morphologies and EMW absorption properties were investigated in detail. This work provides a lead for designing dielectric-magnetic absorbers via a facile method. Moreover, the as-synthesized Fe_3_O_4_/ZnO core–shell structured nanocomposites exhibited an enhanced absorption property, which may be expected to be useful in building a novel platform in advanced EMW absorbers.

## 2. Materials and Methods 

### 2.1. Materials

Ferric chloride (FeCl_3_·6H_2_O), sodium citrate (Na_3_C_6_H_5_O_7_·2H_2_O), sodium acetate (NaOAc), and zinc acetate (Zn(OAc)_2_) were commercially obtained from Aladdin Chemical Reagent, China. Ammonium Hydroxide (NH_3_·H_2_O), ethylene glycol (EG), and absolute ethanol were purchased from Xilong Chemical Reagent Co. Ltd. (Guangzhou, China). All the reagents were used without further purification. Deionized water was produced in our laboratory and used for all experiments. 

### 2.2. Synthesis of Fe_3_O_4_ Nanoparticles

Fe_3_O_4_ nanoparticles (NPs) were prepared by a solvothermal method as reported previously [27]. FeCl_3_·6H_2_O (0.016 mol) and Na_3_C_6_H_5_O_7_·2H_2_O (0.004 mol) were dissolved in EG (70 mL) under magnetic stirring. Then, NaOAc (0.005 mol) was slowly introduced into the mixture solution, generating a transparent suspension. The resulting solution was then transferred into a Teflon-lined stainless-steel autoclave (100 mL capacity). Subsequently, upon sealing, the autoclave was maintained at 200 °C for 10 h. After cooling down to room temperature, the precipitate was collected by the magnet and washed with absolute ethanol and deionized water several times, then dried in a vacuum oven at 50 °C for 12 h.

### 2.3. Synthesis of Fe_3_O_4_/ZnO Nanocomposites

Fe_3_O_4_/ZnO nanocomposites were prepared through the chemical method of coating the magnetic core (Fe_3_O_4_) with ZnO by co-precipitation of Fe_3_O_4_ with Zn(OAc)_2_ in a basic medium of NH_3_·H_2_O. Briefly, the as-prepared Fe_3_O_4_ NPs (0.25 mmol) were dissolved in deionized water (50 mL), Zn(OAc)_2_ (2 mmol) was dissolved in deionized water (20 mL), then the solutions were mixed together by ultrasonic dispersal for 15 min. Subsequently, the mixed solution was mechanically stirred for 0.5 h. In the meantime, a certain amount of NH_3_·H_2_O was added to the suspension. Then the resultant solution was loaded into a 100 mL Teflon-lined stainless-steel autoclave and kept at 120 °C for 15 h. The resulting bronzing product was collected, washed with absolute ethanol and deionized water several times by centrifugation, and then dried in a vacuum oven at 50 °C overnight. the convenience of discussion, the Fe_3_O_4_/ZnO nanocomposites prepared in 3 mL NH_3_·H_2_O and 2 mL NH_3_·H_2_O will be denoted as sample A and sample B, respectively.

### 2.4. Characterization 

**Figure 1:**
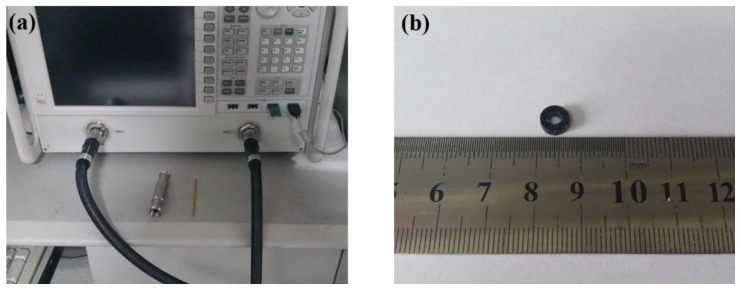
The coaxial waveguide instrumentation (**a**) and the toroidal–shaped sample (**b**).

The crystalline structure and phases of the samples were performed by X-ray diffraction (XRD, Rigaku Denki Co. Ltd., Tokyo, Japan) using a Cu Kα radiation (λ = 0.15418 nm) in a scattering range (2θ) of 10–80° at an accelerating voltage of 40 kV. X-ray photoelectron spectroscopy (XPS) studies were performed using the ESCALAB 250Xi (Thermo Fisher Scientific, Waltham, MA, USA). The morphologies of the as-synthesized samples were characterized by scanning electron microscopy (SEM, JSM-7500F, JEOL, Beijing, China) and transmission electron microscopy (TEM, JEM-2100 microscope with an accelerating voltage of 200 kV, JEOL, Beijing, China). The EM parameters of complex relative permeability (*μ*_r_ = *μ*′ − *jμ*″) and permittivity (*ε*_r_ = *ε*′ − *jε*″) in the frequency range of 2–18 GHz were performed by vector network analyzer, Agilent, N5230A (Agilent Technologies Inc., Santa Clara, CA, USA, as shown in Figure 1a). The as-prepared samples were mixed with paraffin (different mass percentages) and pressed into toroidal-shaped samples (inner diameter *φ*_in_ = 3.04 mm, outer diameter *φ*_out_ = 7.00 mm, as shown in Figure 1b).

## 3. Results and Discussion

**Figure 2:**
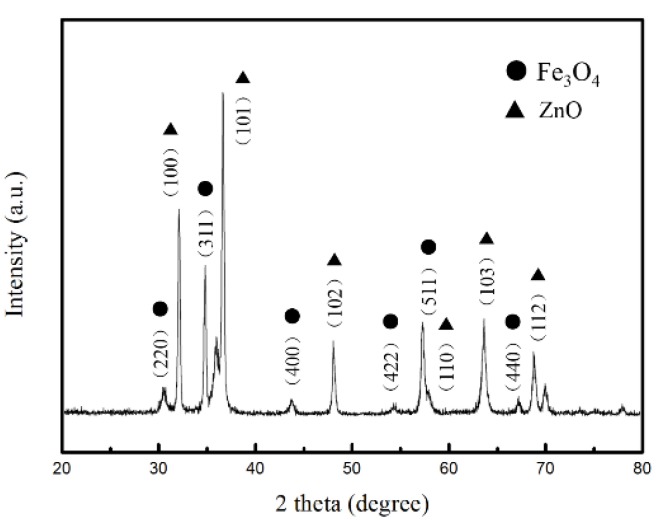
XRD patterns of and Fe_3_O_4_/ZnO composites.

**Figure 3:**
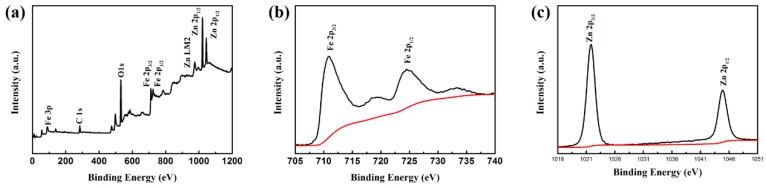
XPS spectra of Fe_3_O_4_/ZnO nanocomposites: (**a**) survey spectrum; (**b**) Fe 2p binding energy spectrum; and (**c**) Zn 2p binding energy spectrum.

To confirm the phases and structures of the as-prepared samples, the corresponding XRD pattern of Fe_3_O_4_/ZnO composites is shown in Figure 2. As for Fe_3_O_4_/ZnO composites, the existence of major diffraction peaks corresponding to the (220), (311), (400), (422), (511), and (440) planes can be observed. These planes can be readily indexed to standard cards of JCPDS No.88-0866, revealing that the crystallinity of Fe_3_O_4_ remains unchanged after coating. Six diffraction peaks were assigned to the (100), (101), (102), (110), (103), and (112) planes, which is consistent with ZnO (JCPDS No.36-1451). Therefore, the XRD patterns confirmed the coexistence of Fe_3_O_4_ and ZnO. The surface elemental states of Fe_3_O_4_/ZnO nanocomposites were further analyzed by XPS, and the results are presented in Figure 3. From the typical survey spectrum, the existence of Fe, Zn, C and O elements can be found. In Figure 3b, the high-resolution spectrum of Fe is given; two peaks appeared at 710.9 and 724.3 eV, corresponding to the band energies of Fe 2p_3/2_ and Fe 2p_1/2_, respectively [28]. This indicates the generation of oxide of Fe_(II)_ and Fe_(III)_, which is in good agreement with the literature and is consistent with Fe_3_O_4_ [29]. The existence of the Fe element indicates that the shell of ZnO may be in porous condition. Figure 3c displays the high-resolution spectrum of Zn. The peaks at 1021.8 and 1044.8 eV correspond to Zn 2p_3/2_ and Zn 2p_1/2_, respectively. Hence, the composites are composed of Fe_3_O_4_ and ZnO.

**Figure 4:**
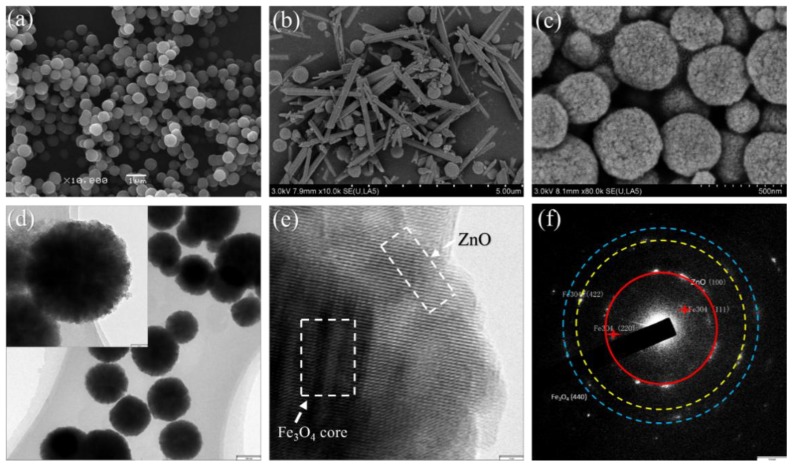
The SEM images of Fe_3_O_4_ (**a**); sample A (**b**); and sample B (**c**); TEM image (**d**); HRTEM image (**e**) of sample A and SAED pattern (**f**) of sample B, respectively.

The scanning electron microscopy (SEM) image of Fe_3_O_4_ NPs is shown in Figure 4a. It can be seen that Fe_3_O_4_ NPs have a relatively uniform spherical shape, and the Fe_3_O_4_ NPs with smooth surfaces have diameters in the range of 250–300 nm. Figure 4b,c shows the as-synthesized Fe_3_O_4_/ZnO nanocomposites under different experimental conditions. Sample A is shown in Figure 4b; it is visible that the products had a disorderly composition comprising a few scattered Fe_3_O_4_ NPs and short ZnO nanorods. The presence of disordered nanorods and NPs suggests that ZnO particles failed to generate chemical bonds with the Fe_3_O_4_ NPs and grew into short rod shapes alone with the introduction of a larger amount of ammonium hydroxide (3 mL). When the amount of ammonium hydroxide was reduced to 2 mL (sample B) in the mix solution, the product exhibited a spherical shape (Figure 4c), and the diameters were a bit larger than those of the Fe_3_O_4_ NPs in Figure 4a. We deduced that Fe_3_O_4_ NPs were uniformly covered by the ZnO shells in a spherical shape. The magnetic NPs are utilized as a seed-mediated growth mechanism to grow a layer of ZnO on their surfaces, thus making the surface much rougher than the naked Fe_3_O_4_ NPs. The morphology and distribution of the Fe_3_O_4_/ZnO core–shell structured nanocomposites are clearly recognizable from the low-magnification TEM image in Figure 4d. It can be discerned that the nanocomposites are nearly spherical in shape, with a diameter distribution of 280–330 nm, which is consistent with the SEM image in Figure 4c. The high-resolution TEM image of one typical core–shell structured Fe_3_O_4_/ZnO composite is demonstrated in Figure 4e. The distinction between the transparent boundary and the dark core confirms the growth of ZnO on the Fe_3_O_4_ NP. It can be observed that a thin layer of ZnO is growing on the edge of Fe_3_O_4_ NP at a thickness of ~15 nm. Figure 4f also presents the diffraction profile generated by the inserted SAED pattern and confirms the structure of ZnO and Fe_3_O_4_. The SEM and TEM results clearly indicate that the nanocomposites possess a core-shell type structure, and that the inner Fe_3_O_4_ NPs cores are successfully wrapped with the uniformed ZnO shells.

**Figure 5:**
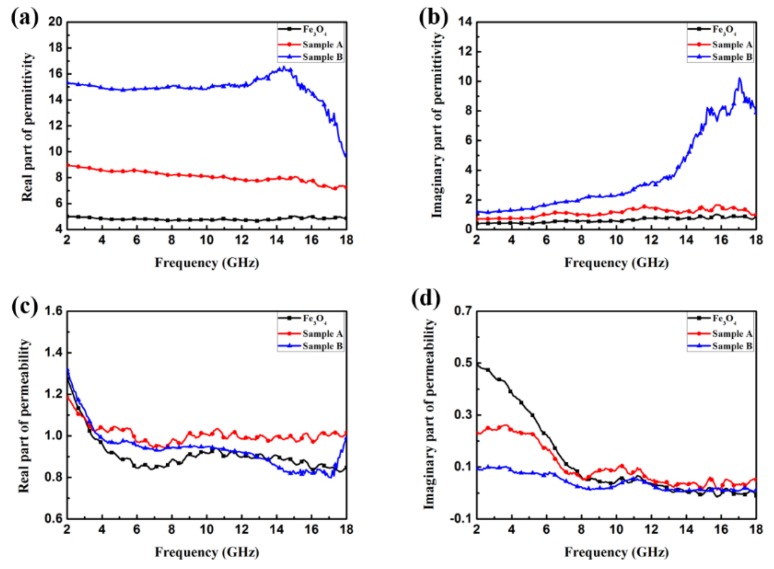
Frequency dependence on the (**a**) real part and (**b**) imaginary part of relative complex permittivity; (**c**) real part and (**d**) imaginary part of relative complex permeability.

In order to explore the microwave absorption properties of Fe_3_O_4_/ZnO nanocomposites, the relative complex permeability (*μ*_r_ = *μ*′ − j*μ*″) and relative permittivity (*ε*_r_ =*ε*′ − j*ε*″) were measured by a vector network analyzer in the frequency range of 2–18 GHz. The measured samples were prepared by uniformly mixing with paraffin (in mass fractions of 30%, 50%, and 70%) at 85 °C, pressed into toroidal-shaped samples. According to transmission line theory, EM properties can be evaluated based on the relative complex permeability (*μ*_r_ = *μ*′ − j*μ*″) and relative permittivity (*ε*_r_ =*ε*′ − j*ε*″). *μ*′ and *ε*′ represent the ability to store EM energy, whereas *μ*″ and *ε*″ represent the inner dissipation of EM energy, which originates from the relaxation and resonance mechanisms. The relative complex permittivity (*ε*′, *ε*″) and relative complex permeability (*μ*′, *μ*″) of Fe_3_O_4_, sample A and sample B measured in the frequency range of 2–18 GHz are plotted in Figure 5a–d. From Figure 5a,b, it can be found that *ε*′ of Fe_3_O_4_ is in the range of 4.67–5.03, and *ε*″ of Fe_3_O_4_ is in the range of 0.40–1.02. After being composited with ZnO, the values of *ε*′ and *ε*″ show a sharp growth. As for sample A, the values of *ε*′ and *ε*″ are in the range of 7.12–8.94 and 0.70–1.67, respectively. Meanwhile, the values of *ε*′ and *ε*″ raise to the range of 9.62–16.60 and 1.17–10.23, respectively, after the Fe_3_O_4_ NPs were coated with the ZnO shell (sample B). The values of *ε*′ and *ε*″ for sample B fluctuate more and exhibit great change in the main measuring frequency region. As shown in Figure 5a, the *ε*′ of sample B presents a declining trend with increasing frequency, while the trend of *ε*″ is the contrary, and some peaks appear in the high frequency region. The curves of *ε*′ and *ε*″ indicate that the ZnO shell can greatly improve the dielectric properties of the material. Figure 5c,d shows the relative complex permeability of the three materials. As for Fe_3_O_4_ NPs, the value of *μ*′ drops sharply from 1.27 in the frequency range of 2–6 GHz, and then shows a fluctuating trend versus the changing frequency, and the Fe_3_O_4_/ZnO (sample A and B) composites display a similar variation trend throughout the entire measured frequency range. This phenomenon may result from the eddy current effect. Compared to the value of *μ*″, differences in the *μ*″ values of Fe_3_O_4_ and Fe_3_O_4_/ZnO composites are distinguished at low frequency. Meanwhile, in the range of 8–18 GHz, the *μ*″ curves of Fe_3_O_4_ and Fe_3_O_4_/ZnO composites change to become similar; one *μ*″ value peak of sample B is observed at ~11 GHz, which may be attributable to the dissipation of EM energy. Furthermore, it is noticed that negative values of *μ*″ occur in the high frequency range due to calibration or sensitivity issues of the experimental set-up. 

The theoretical reflection loss (RL) of the composite absorber at different thicknesses was calculated using the following equations [30,31,32]:

(1) $$RL=20\;log|\left(Z_{in}-Z_{0}\right)/\left(Z_{in}+Z_{0}\right)|$$

(2) $$Z_{in}=Z_{0}\sqrt{\frac{\mu_{r}}{\varepsilon_{r}}}tanh\left(j\frac{2\pi fd\sqrt{\mu_{r}\varepsilon_{r}}}{c}\right)$$

Here, *Z*_0_ is the impedance of free space, *Z*_in_ is the normalized input impedance of the absorber, *d* is the thickness, *C* is the velocity of EMW in free space, and *f* is the frequency of the incident wave. RL values of −10 dB and −20 dB correspond to 90% and 99% attenuation of the incident EMW energy, and the frequency range where RL is smaller than −10 dB is defined as the effective absorption bandwidth. 

**Figure 6:**
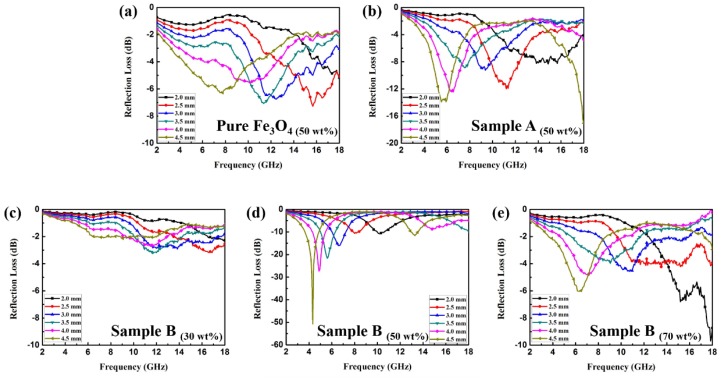
RL curves of paraffin samples containing 50 wt % Fe_3_O_4_ (**a**) and sample A (**b**); RL curves of paraffin samples containing 30 wt % (**c**); 50 wt % (**d**) and 70 wt % (**e**) sample B, respectively.

**Figure 7:**
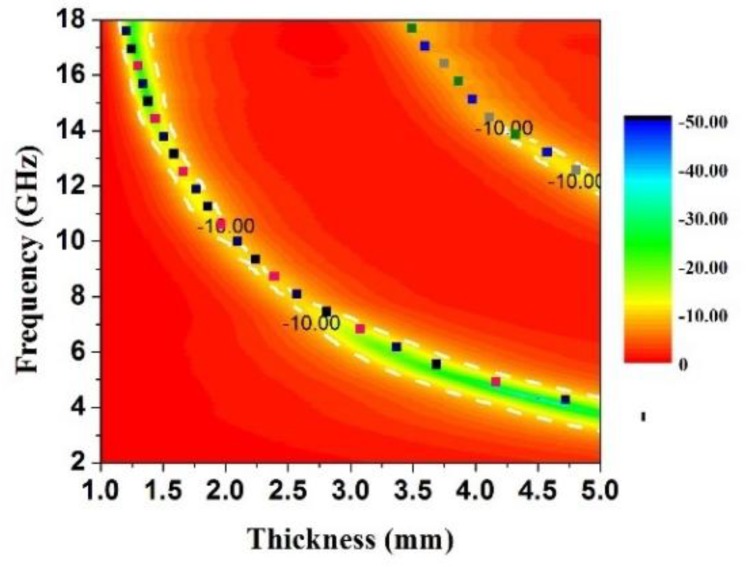
RL 2-D contour map representations in the frequency range of 2–18 GHz loaded with 50 wt % of sample B.

Figure 6a–e shows the plots of RL versus the frequency of the Fe_3_O_4_ NPs and two samples of Fe_3_O_4_/ZnO composites at different thicknesses. As for Fe_3_O_4_ NPs, the minimum RL of −7.28 dB is observed at 15.68 GHz with the thickness of 2.5 mm, which indicates that the naked Fe_3_O_4_ NPs have a weak EMW absorption property. Furthermore, with the doping of the dielectric component, the EMW absorption property of sample A can be improved slightly. As shown in Figure 6b, the minimum RL is −13.91 dB at 5.52 GHz with the thickness of 4.5 mm. This is because the ZnO particles failed to generate chemical bonds with the Fe_3_O_4_ NPs and grew into short rod shapes alone; therefore, the composites were unable to obtain a good impedance match and interfacial polarization. As for the sample B loaded with 30 wt % (Figure 6c), because of the high dispersion in the paraffin matrix, the Fe_3_O_4_/ZnO nanoparticles failed to generate conductive interconnections, so the EMW absorption performance did not show an enhancement in comparison to pure Fe_3_O_4_. It is noticed that sample B loaded with 50 wt % shows an enhanced EMW absorption property (Figure 6d). Specifically, the minimum RL value of −50.79 dB can be achieved at 4.38 GHz with the thickness of 4.5 mm. Based on the results of Figure 4d–f, we deduced that the incorporation of the dielectric ZnO into the Fe_3_O_4_ NPs may generate a high dielectric constant and loss due to the effective interfaces between the dielectric and magnetic materials, giving them an advantage in terms of matching complex permittivity and permeability. The enhanced EM absorption properties benefit from the uniform core–shell structures, which induce an intensification of interfacial polarization. It is worth noting that the core–shell structured Fe_3_O_4_/ZnO composites are able to achieve an enhanced absorption property in both low and high frequency bands; such dual absorption regions are also competitive in comparison to other materials. It can be observed from Figure 6d that the minimum RL values all shift toward the lower frequency region with increasing thickness, which can be explained by the quarter-wavelength match principle [18]:

(3) $$t_{m}=\frac{n\lambda }{4}=\frac{nc}{4f_{m}\sqrt{\left(\left|\varepsilon r\mu r\right|\right)}}\left(n=1,3,5\ldots \right)$$

where *t_m_* is the absorber thickness, *μ_r_* is the complex permeability at *f_m_*, and *ε_r_* is the complex permittivity at *f_m_*. The frequency dependence of *t_m_* (*n* = 1, 3) is calculated and plotted on the contour maps in Figure 7. It can be noticed that all the points of RL_min_ lie on the curves of *t_m_* for sample B. Thus, it is demonstrated that the quarter-wavelength match principle is an effective tool that provides a crucial guide in the thickness design of absorbers.

Typically, the magnetic loss is implied by the imaginary part of permeability and mainly originates from hysteresis loss, domain wall displacement, natural resonance, and eddy current resonance. In general, hysteresis loss is mainly caused by the time lag of the magnetization vector behind the external EM-field vector and will always be negligible in a weak applied field, while domain wall resonance loss takes place in the MHz frequency range. The following equation is used to determine whether eddy currents contribute to the magnetic loss [33]:

(4) $$C_{0}=\mu^{\prime \prime }{\left(\mu^{\prime }\right)}^{-2}f^{-1}$$

**Figure 8:**
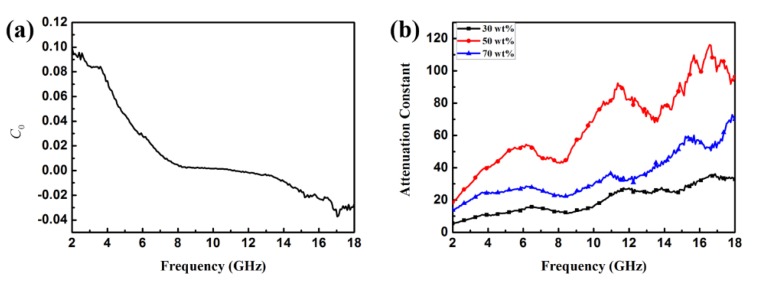
Frequency dependence of *C*_0_ (**a**) and values of attenuation constant of α (**b**) of sample B in the range of 2–18 GHz.

If magnetic loss only stems from the eddy current, *C*_0_ should be equal to a constant value 2*πμ*_0_*d*^2^*σ* (*d* is the thickness of the MAMs, *σ* is the electrical conductivity, and *μ*_0_ is the permeability in a vacuum) and would be independent of frequency; if not, the magnetic loss is ascribed to natural resonance. From Figure 8a, we find that the value of *C*_0_ varies with the frequency and presents a sharp declining tendency in the frequency range of 2–8 GHz. However, when the frequency is in the range of ~8–12 GHz, the value of *C*_0_ closes to a constant. Based on this phenomenon, it can be concluded that magnetic loss results from the natural and exchange resonance and the eddy current effect. 

**Figure 9:**
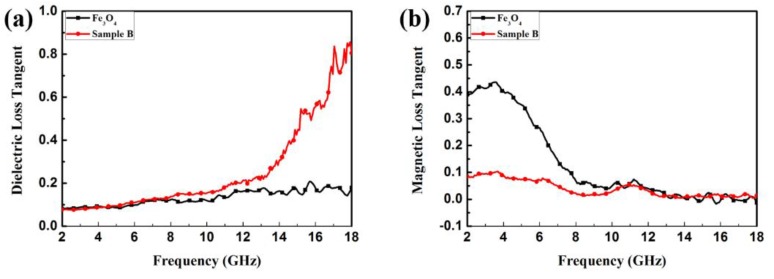
Dielectric loss tangent (**a**) and magnetic loss tangent (**b**) of the Fe_3_O_4_ and Fe_3_O_4_/ZnO (sample B), respectively.

According to transmission line theory, EMW absorption properties can be expressed by the attenuation constant of α. The attenuation constants of sample B with 30, 50, and 70 wt % were calculated using the following expression [34,35]:

(5) $$\alpha =\frac{\sqrt{2}\pi f}{c}\times \sqrt{\left(\mu^{\prime \prime }\varepsilon^{\prime \prime }-\mu^{\prime }\varepsilon^{\prime }\right)+\sqrt{{\left(\mu^{\prime \prime }\varepsilon^{\prime \prime }-\mu^{\prime }\varepsilon^{\prime }\right)}^{2}+{\left(\mu^{\prime }\varepsilon^{\prime \prime }+\mu^{\prime \prime }\varepsilon^{\prime }\right)}^{2}}}$$

where *c* is the velocity of light in a vacuum. Figure 8b shows the plot of the attenuation constant of α versus frequency. It can be seen that the sample with 50 wt % filler loading has the largest value of α; thus, we supposed that the sample with 50 wt % filler loading possesses greater EMW attenuation and impedance matching than the other samples. Figure 9 shows the dielectric loss (tan*δ_ε_* = *ε*″/*ε*′) and magnetic tangent loss (tan*δ_μ_* = *μ*″/*μ*′) of Fe_3_O_4_/ZnO and Fe_3_O_4_ NPs, respectively. tan*δ_ε_* and tan*δ_μ_* are two possible contributors for EMW absorption, and are commonly used to describe material loss capacity. Therefore, we calculated the tangent loss based on the data in Figure 5. Specifically, sample B has a higher tan*δ_ε_* value than the naked Fe_3_O_4_ NPs, indicating that the ZnO shell obviously improves the dielectric properties of the composites. Additionally, Figure 7 clearly shows that the magnetic loss factor (tan*δ_μ_*) is much higher than the dielectric loss factor (tan*δ_ε_*) in the low frequency range (~2–7 GHz), which indicates that magnetic loss plays a vital role in EMW absorption in this region. Meanwhile, in the high frequency range (~8–12 GHz), the value of tan*δ_ε_* is higher than tan*δ_μ_*, which indicates that dielectric loss is the main loss in this frequency region. Such a complementarity between dielectric loss and magnetic loss demonstrates the Fe_3_O_4_/ZnO composites to possess promising EMW absorption properties. 

In Table 1, the recently reported EMW absorption performances of typical Fe_3_O_4_ material-based composites, as well as the Fe_3_O_4_/ZnO composites prepared in this work, have been plotted. In comparison with the reported composites in Table 1, it can be observed that the Fe_3_O_4_/ZnO composites have a wide effective absorption bandwidth and a promising negative RL value among these composites. It can be concluded that the as-fabricated Fe_3_O_4_/ZnO nanocomposites with enhanced EMW absorption properties confirm the presence of an efficient complementarity between magnetic and dielectric loss. The above-mentioned advantages indicate that this special core–shell structured absorber is able to meet the requirements of ideal MAMs.

## 4. Conclusions

In summary, Fe_3_O_4_/ZnO nanocomposites were synthesized via a chemical method of coating magnetic cores (Fe_3_O_4_) with ZnO by co-precipitation of Fe_3_O_4_ with zinc acetate in a basic medium of ammonium hydroxide, and the morphology and the microwave absorption properties were investigated in detail. It is suggested that the amount of ammonium hydroxide plays a key role in controlling the morphologies of the composites, and the SEM and TEM results further confirmed that ZnO shell generated chemical bonds with the Fe_3_O_4_ NPs. Owing to the core–shell structure, an efficient complementary balance was achieved between dielectric loss and magnetic loss. Moreover, the enhanced microwave absorption properties benefitted from the core–shell structure, which induces intensified interfacial polarization. Specifically, the minimum RL value of −50.79 dB can be achieved at 4.38 GHz when the thickness is 4.5 mm. The mechanism of designing neoteric structures with magnetic and dielectric materials in order to broaden the effective absorption bandwidth would open up a promising domain in designing composites with high EM absorption performance. As a result, our Fe_3_O_4_/ZnO nanocomposites are expected to form a novel platform for advancing EMW absorbers.

## Figures and Tables

**Table 1:** EMW absorption performances of typical Fe_3_O_4_-based composites reported in this work and recent literature.

Sample	wt (%)	Optimum Frequency (GHz)	Minimum RL Value (dB)	Ref.
SnO_2_/Fe_3_O_4_/MWCNTs	70	10.90	−42.00	[36]
Fe_3_O_4_/SiO_2_/rGO	20	9.70	−26.60	[37]
Fe_2_O_4_/MnO_2_	40	16.80	−41.50	[38]
Fe_3_O_4_@C	66.7	16.20	−22.60	[39]
FePc-Fe_3_O_4_-BF	75	5.90	−31.10	[40]
Fe_3_O_4_/ZnO	50	4.38	−50.79	This work
